# Coexistence of Multiple Endocrine Neoplasia Type 2B and Chilaiditi Sign: A Case Report

**DOI:** 10.1155/2012/360328

**Published:** 2012-10-17

**Authors:** Deniz Çetin, Mustafa Ünübol, Aykut Soyder, Engin Güney, Adil Coşkun, Serdar Özbaş, Alparslan Ünsal, Muhan Erkuş

**Affiliations:** ^1^Department of Internal Medicine, Adnan Menderes University Faculty of Medicine, 09100 Aydin, Turkey; ^2^Division of Endocrinology and Metabolism, Adnan Menderes University Faculty of Medicine, 09100 Aydin, Turkey; ^3^Department of General Surgery, Adnan Menderes University Faculty of Medicine, 09100 Aydin, Turkey; ^4^Division of Gastroenterology, Adnan Menderes University Faculty of Medicine, 09100 Aydin, Turkey; ^5^Department of Radiology, Adnan Menderes University Faculty of Medicine, 09100 Aydin, Turkey; ^6^Department of Pathology, Adnan Menderes University Faculty of Medicine, 09100 Aydin, Turkey

## Abstract

We present a 15-year-old female patient with medullary thyroid carcinoma, marfanoid habitus, and mucosal ganglioneuromatosis. Our case had a *RET* protooncogene mutation ser836 polymorphism in exon 14 and ser904 polymorphism in exon 15. Our patient is thought to be atypical MEN2B due to the absence of M918T or A883F mutations. Chilaiditi sign is an incidental radiographic finding of a usually asymptomatic condition in which a part of intestine is located between the liver and diaphragm; however, the term “Chilaiditi syndrome” is used for symptomatic hepatodiaphragmatic interposition. The patient had no symptoms as abdominal pain, constipation, diarrhea, or emesis. Incidentally, Chilaiditi sign was diagnosed with chest radiograph and thoracoabdominal CT. Our case is the first in the literature indicating the coexistence of Chilaiditi sign and MEN2B.

## 1. Introduction

Multiple endocrine neoplasia type 2B (MEN2B) is a rare autosomal dominant syndrome characterized by medullary thyroid carcinoma (MTC), pheochromocytoma, marfanoid habitus, and mucosal/intestinal ganglioneuromatosis. Gastrointestinal ganglioneuromatosis is the predominant etiology of gastrointestinal symptoms in patients with MEN2B [1]. Chilaiditi sign is an incidental radiographic finding of a usually asymptomatic condition in which a part of intestine is located between the liver and diaphragm; however, the term “Chilaiditi syndrome” is used for symptomatic hepatodiaphragmatic interposition [2]. We detected Chilaiditi sign on chest radiograph and thoracoabdominal CT of our patient with MEN2B. In the literature, Chilaiditi sign was not previously described in MEN2B. Our case is the first case in which MEN2B is accompanied by Chilaiditi sign.

## 2. Case Presentation

**Figure 1:**
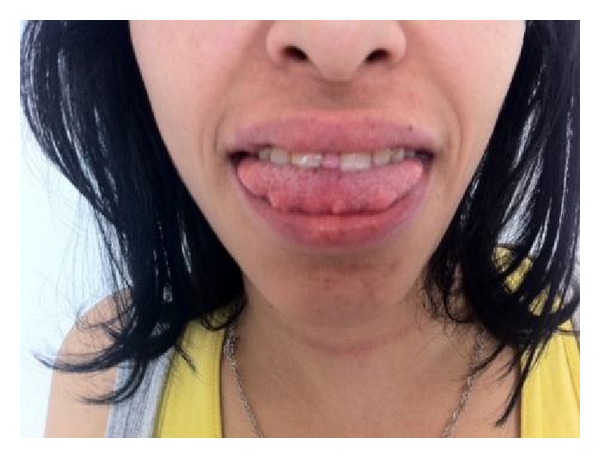
Mucosal ganglioneuromatosis.

**Figure 2:**
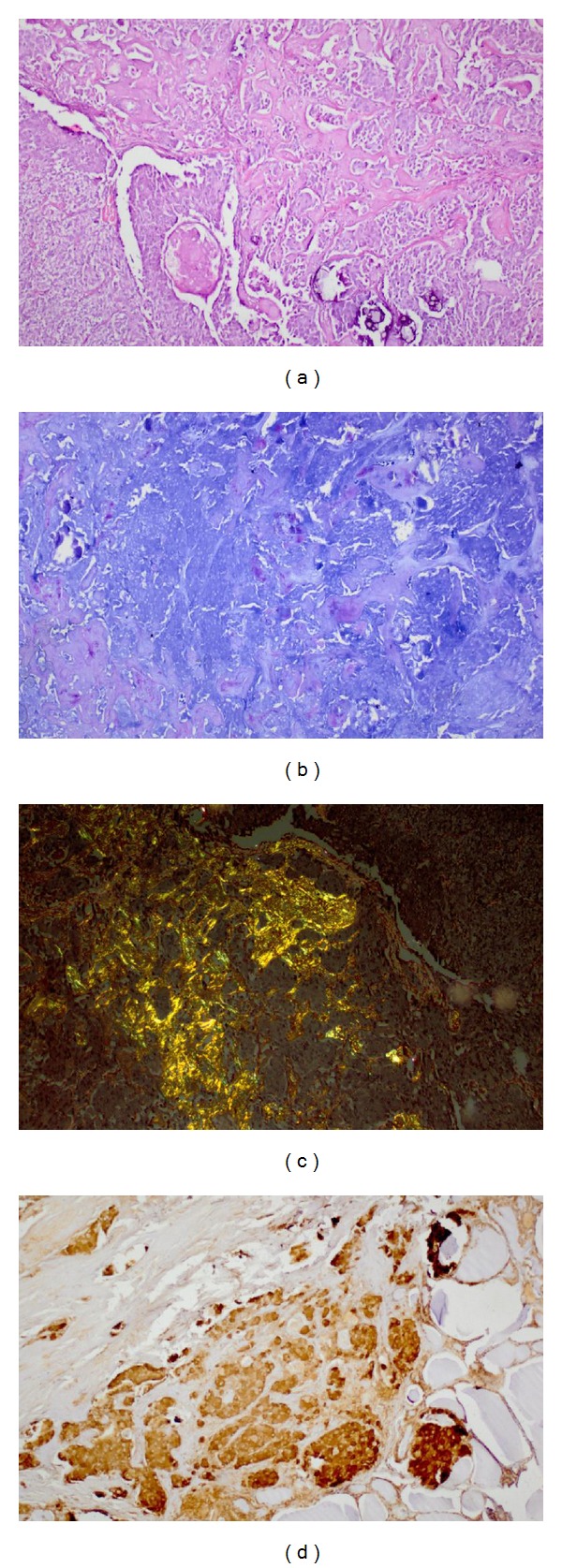
The immunohistochemical staining of tumor sections (a) Hematoxylin and eosin reveal insular and follicular growth pattern characteristics of spindle cells. (b) and (c) The deposition of amyloid is detected in some foci with crystal violet and congo red. (d) The staining with calcitonin.

**Figure 3:**
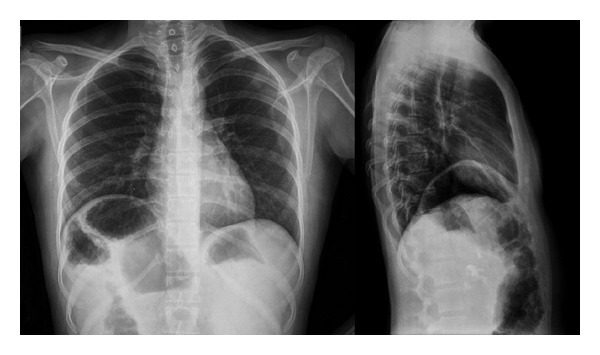
Colonic interposition between the liver and right hemidiaphragm.

**Figure 4:**
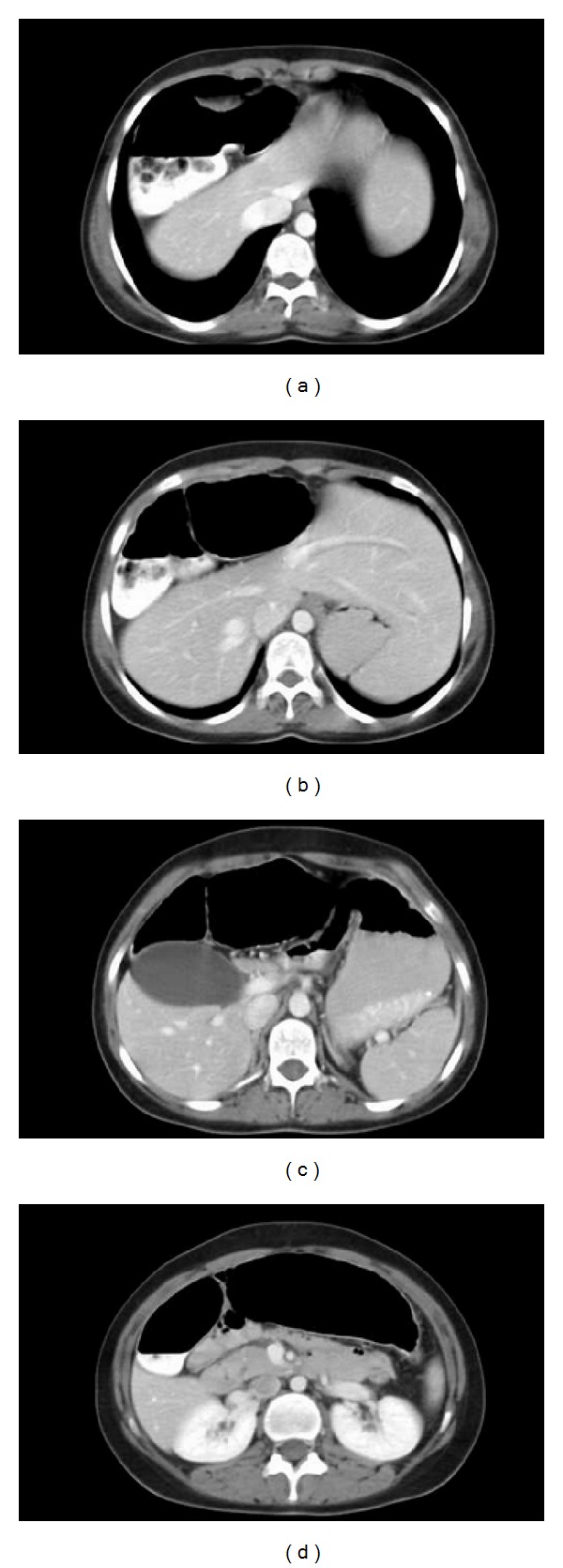
Colonic interposition between the liver and right hemidiaphragm. (a) and (b) The colonic segment which displaced the liver towards the left side wall of the abdomen. (c) Hydropic gallbladder and normal adrenal glands. (d) The head of pancreas is normal.

A 15-year-old female was admitted with complaint of painless swelling of the neck. Physical examination findings were marfanoid habitus, bilateral palpable thyroid nodules, and numerous yellowish-white, sessile, painless nodules on the tongue (Figure 1). Laboratory findings are TSH 2.29 mU/mL (reference range 0.27–4.2 mU/mL), free T4 1.30 ng/dL (reference range 0.93–1.7 ng/dL), free T3 3.42 pg/mL (reference range 1.8–4.6 pg/mL), antithyroglobulin antibody 15.29 IU/mL (reference range 0–115 IU/mL), parathormone 53.95 pg/mL (reference range 15–65 pg/mL), calcitonin 528 pg/mL (reference range 0–11.5 pg/mL), and CEA 29.2 mcg/L (reference range 0–2.5 mcg/L). Ultrasound imaging of the thyroid gland revealed bilateral, hyperecogenic nodules containing foci of microcalcification with the largest diameter of 23 mm. The patient was thought to be MTC. Pheochromocytoma was excluded by laboratory findings including urinary VMA, metanephrine, and normetanephrine within normal range. The patient underwent total thyroidectomy and central lymph node dissection. In the surgical specimen, tumor cell clusters were observed 2.3 cm at the left lobe, 1 cm at the right lobe. Metastasis was detected in 7 of 9 nodes resected. The immunohistochemical staining of tumor sections revealed diffuse positive staining with chromogranin, NSE, calcitonin, CEA, synaptophysin, and keratin. There was no staining with thyroglobulin. The deposition of amyloid which was stained with crystal violet and congo red was detected in some foci. Spindle cells showed insular and follicular growth pattern characteristics (Figure 2). Tests for *RET* protooncogene in exon 14 (ser836 polymorphism) and exon 15 (ser904 polymorphism) were positive, but exons 10, 11, 13, and 16 were negative. *RET* protooncogene examination of the patient's family members showed that father and brother had ser904 polymorphism in exon 15; exon 16 was negative. There was no evidence of metastasis in the postoperative evaluation including cervical ultrasound, thoracic and abdominal CT, PET/CT, and radionuclide bone scan. Serum calcitonin levels were monitored periodically. Chest radiograph and thoracoabdominal CT showed colonic interposition between the liver and right hemidiaphragm (Figures 3 and 4). The patient had no symptoms as abdominal pain, constipation, diarrhea or emesis. Chilaiditi sign was diagnosed incidentally. Rectoscopy was performed because the patient did not admit colonoscopy. Rectal biopsy was normal and there was no mucosal ganglioneuromatosis.

## 3. Discussion

MEN2B typically manifests in the first year of life with mucosal neuromas, intestinal ganglioneuromas, and marfanoid habitus as a result of de novo mutation [3]. The prevalence of MEN2 has been estimated at 1 : 35000 [4]. MEN2B generates <10% of MEN2 syndromes with a higher mortality rate [5]. All MEN2 variants are based on germline mutation in the *RET *gene and MEN2B is the most aggressive of these variants [6]. In MEN2B patients, ≥95% of cases present the M918T mutation in exon 16, 2%-3% of cases present the A883F mutation in exon 15 [7, 8]. *RET* mutations at codons 805, 806, and 904 in cis configuration with the P.V804 M mutation have also been reported [9–11]. single-nucleotide Polymorphisms (SNPs) within the *RET* oncogene have been reported (G691S, L769L, S836S, and S904S), and the modifying role of the clinical phenotype of MEN2 has been discussed [3, 12]. Our case had a *RET* protooncogene mutation ser836 polymorphism in exon 14 and ser904 polymorphism in exon 15. Our patient is thought to be atypical MEN2B due to the absence of M918T or A883F mutations. Gastrointestinal ganglioneuromatosis is the predominant etiology of gastrointestinal symptoms in patients with MEN2B, resulting in bowel tone decrease, distention, segmental dilatation, and megacolon. The most reported symptoms were constipation and intermittent diarrhea [1, 13]. Gastrointestinal symptoms usually manifest in infancy and present before symptoms of extraintestinal endocrine abnormalities [1, 13–15]. Chilaiditi sign is an incidental radiographic finding of a usually asymptomatic condition in which a part of intestine is located between the liver and diaphragm; however, the term “Chilaiditi syndrome” is used for symptomatic hepatodiaphragmatic interposition [2]. It was first described by Demetrious Chilaiditi in 1910. The incidence of this syndrome is 0.025–0.28% [16]. Megacolon, abnormal colonic motility, eventration of diaphragm, phrenic nerve injury, cirrhosis, a congenital etiology, laxity of the suspensory ligaments, obesity, and previous surgery are possible factors that may lead to this syndrome [2]. Nausea, vomiting, constipation, abdominal distention, and pain are gastrointestinal symptoms of the Chilaiditi syndrome. Respiratory distress, cardiac arrhythmia, acute intestinal obstruction, and volvulus of the colon are possible complications [17]. Conservative management includes bed rest, nasogastric and/or rectal decompression, and liquid replacement. Surgery may be required in patients with persistent pain, refractory ileus, colonic volvulus, or bowel ischemia [17, 18]. We wanted to emphasize the coexistence of MEN2B and Chilaiditi syndrome in differential diagnosis due to the similarity of symptoms that can be seen in Chilaiditi syndrome and MEN2B related to gastrointestinal ganglioneuromatosis. In our patient, gastrointestinal ganglioneuromatosis was not considered because she had no gastrointestinal complaints. Chilaiditi sign has not been described in patients with MEN2B. Also, our case is the first in the literature indicating the coexistence of Chilaiditi sign and MEN2B.
